# Family Medicine Education at a Rural Hospital in Japan: Impact on Institution and Trainees

**DOI:** 10.3390/ijerph18116122

**Published:** 2021-06-06

**Authors:** Ryuichi Ohta, Yoshinori Ryu, Chiaki Sano

**Affiliations:** 1 Community Care, Unnan City Hospital, Shimane 699-1221, Japan; yoshiyoshiryuryu.hpydys@gmail.com; 2 Department of Community Medicine Management, Faculty of Medicine, Shimane University, Shimane 690-0823, Japan; sanochi@med.shimane-u.ac.jp

**Keywords:** family medicine, educational curriculum, scope of practice, unlearning, rural community hospital

## Abstract

Family medicine is vital in Japan as its society ages, especially in rural areas. However, the implementation of family medicine educational systems has an impact on medical institutions and requires effective communication with stakeholders. This research—based on a mixed-method study—clarifies the changes in a rural hospital and its medical trainees achieved by implementing the family medicine educational curriculum. The quantitative aspect measured the scope of practice and the change in the clinical performance of family medicine trainees through their experience of cases—categorized according to the 10th revision of the International Statistical Classification of Disease and Related Health Problems. During the one-year training program, the trainees’ scope of practice expanded significantly in both outpatient and inpatient departments. The qualitative aspect used the grounded theory approach—observations, a focus group, and one-on-one interviews. Three themes emerged during the analysis—conflicts with the past, driving unlearning, and organizational change. Implementing family medicine education in rural community hospitals can improve trainees’ experiences as family physicians. To ensure the continuity of family medicine education, and to overcome conflicts caused by system and culture changes, methods for the moderation of conflicts and effective unlearning should be promoted in community hospitals.

## 1. Introduction

Family medicine is vital in medical institutions in Japan as its society ages [1]. Family physicians have the ability to comprehensively handle various problems in patients across multiple categories of diseases [1,2]. Additionally, as social and psychological issues can cause medical problems, family physicians approach patients using the biopsychosocial approach, focusing on biology as well as social and psychological contexts [3,4]. The allocation of family physicians can alleviate multimorbidity and polypharmacy in older patients [5,6]. An increase in the number of family physicians is essential for globally aging societies, creating impetus to increase the number of family physicians by constructing educational systems with family physicians in medical institutions and communities [7,8,9]. In the Japanese context, national insurance provides medical care with the free access system [10]. Patients in Japan can go to any medical institution if they exhibit any symptom, which may cause fragmentation of care regarding issues of multimorbidity and polypharmacy [11,12]. Family medicine offers a solution to these problems.

Educational systems for family medicine are needed, the establishment of which involves various considerations. The application of new educational systems requires effective communication with stakeholders such as deans, medical educators, and other professionals [13,14]. Additionally, educational environments can be affected by the context and culture of medical institutions, such as those in rural and urban settings [15,16]. The application of family medicine education curriculum to institutions can serve to modify the working conditions of professionals [16,17]. Although the establishment of educational systems for family medicine is critical, issues related to contextual factors should be considered. The educational systems and contextual factors need to be adapted through continual dialog and negotiation among staff in participating institutions.

The practical application of family medicine education to institutions should be promoted, and the methods should be clarified. Family medicine education curriculum can provide effective education for family physicians, who can contribute to comprehensive healthcare from pediatrics to geriatrics [18]. Especially in rural areas, where healthcare resources are lacking, the education of family physicians is critical to ensure the sustainability of rural health care [19,20]. However, in the current literature, the process of implementing family medicine education, including difficulties and challenges, has not been clarified. Further, there is no existing research that documents the changes in rural hospitals resulting from the implementation of family medicine education. Clarification of the processes of implementing educational systems in rural hospitals can be valuable for family physicians; such processes can be investigated using the action research method. A revision of the difficulties and challenges can facilitate the implementation of family medicine education, contributing to the education of more family physicians who can provide comprehensive care in aging societies. Thus, the purpose of this research is to clarify the changes in rural hospitals and medical trainees incited by the implementation of educational curriculum for family medicine, using a mixed-method study.

## 2. Materials and Methods

This mixed-method research was implemented in collaboration with the stakeholders of family medicine education in a rural hospital using the data of patients who were cared for by the trainees. This research was conducted for two years, from 1 April 2020 to 31 March 2021. Ethnography and interviews were also undertaken to clarify how the application of the educational curriculum affected a rural hospital. The researchers were participatory observers, who educated the trainees, and discussed the application with stakeholders working in a rural hospital. Additionally, the clinical performance of the trainees before and after the education—in the first and final months, respectively—was compared to assess the improvement of their competencies. The comparison was based on the expanse of the scope of practice and assessment of interprofessional collaboration.

### 2.1. Setting

Unnan, located in the southeast area of the Shimane Prefecture, is one of the most rural cities in Japan. In 2020, the total population of Unnan was 37,638 (18,145 males and 19,492 females), with an aging rate of 39%, which is expected to reach 50% by 2025. Each family lives separately, but autonomous communities have been established for the sustainability of neighborhoods [21]. In Unnan, there are 16 clinics, 12 home care stations, 3 visiting nurse stations, and only 1 public hospital (Unnan City Hospital, Unnan, Japan). At the time of the study, Unnan City Hospital had 281 beds, comprising 160 acute care beds, 43 comprehensive care beds, 30 rehabilitation beds, and 48 chronic care beds. There were 14 medical specialists, and the nurse-to-patient ratio was 1:10 for acute care, 1:13 for comprehensive care, 1:15 for rehabilitation, and 1:25 for chronic care. Hospital staff was comprised of 27 physicians, 197 nurses, 7 pharmacists, 15 clinical technicians, 37 therapists, 4 nutritionists, and 34 clerks. Three physicians in the hospital specialized in family medicine/primary care and treated patients with multiple diseases in both inpatient and outpatient situations. All were engaged in the education of medical students and residents [22,23].

### 2.2. Participants

The participants were all staff members involved in medical care or medical trainees in Unnan City Hospital. This study involved medical educators, family medicine trainees, medical students/residents, nurses, pharmacists, therapists, social workers, clinical technicians, nutritionist, and clerks. They collaborated on each patient’s care in the hospital. The patients who were cared for by the family medicine trainees were included in the assessment of the trainees’ scope of practice.

### 2.3. Educational Curriculum

This educational curriculum is based on the Japanese Primary Care Association’s board of family medicine, which was developed based on the world standard of education of family medicine [24]. In this curriculum, trainees experience various clinical situations and patients. In the first year, trainees work at a community hospital (Unnan City Hospital) for one year and experience typical diseases in both inpatient and outpatient situations. They also work at a rural clinic to learn home care and community-oriented primary care for six months. To broaden the scope of practice regarding internal medicine, pediatrics, and emergency medicine, they work at a general hospital. Each clinical setting has medical teachers. Trainees learn content through cognitive apprenticeship, legitimate peripheral participation, and continuous reflection with medical teachers. The formative and summative assessments of the learners are accomplished using the Mini-CEX, multiple source feedback, and portfolios. After three years of training, the trainees take a national examination in family medicine and obtain a family physician’s certificate [21]. In this study, the focus is on how medical trainees work at a community hospital and how the hospital is impacted. In the first year, which begins on April 1, medical trainees collaborate with various medical professionals in a community hospital. This curriculum can educate a maximum of three trainees. The curriculum accommodated one trainee in 2018 and 2019, and three in 2020.

### 2.4. Instrumentation

#### 2.4.1. The Performance Data of the Trainees

Mini-CEX and multisource feedback were implemented to assess the trainees’ approaches to patients and other professionals, to improve their performance. To follow-up on their learning, written portfolios about family medicine were checked and provided with feedback. To assess their clinical performance in inpatient and outpatient departments, the demographics of patients taken care of by the trainees and their total number of diseases in inpatient and outpatient departures were measured each month.

#### 2.4.2. Questionnaire Assessing Interprofessional Collaboration with Nurses

In the first year of the training at the community hospital, the medical trainees worked in the inpatient wards, thus, most interprofessional collaborations were with a variety of nurses. The nurses then discussed the abilities of the trainees with their deputy head nurses. To assess the trainees’ interprofessional abilities comprehensively, the questionnaire regarding inter-professional collaboration was provided to deputy head nurses in the hospital to inquire about trainees’ abilities in interprofessional collaboration in the hospital, based on previous research [25]. The contents included introducing oneself, respect for nurses’ opinions, efficient discussion with nurses, politeness to nurses, respect for nurses as members of the medical team, respecting nurses’ ideas concerning care management, explaining treatment plans to nurses, answering nurses’ questions clearly, conveying treatment plans via oral or electronic medical records to nurses, and apologizing to nurses when they make mistakes. Each category was assessed on a five-point Likert scale. This survey was conducted twice—four months into the program and at the end of this study.

#### 2.4.3. Direct Observation

As medical teachers and participatory researchers, the authors educated the trainees about family medicine and observed their learning and their interactions with other medical and care professionals in the hospitals. The trainees undertook daily reflections and discussed their learning and their difficulties in their workplaces with the authors, particularly learning content and interactions with other professionals. The contents of the observations, reflections, and discussions were written up in field notes.

#### 2.4.4. Focus Groups and One-on-One Interviews

Once a month, focus group discussions were undertaken with the nursing, pharmacy, therapy, and clerical departments. Each session included 3–6 participants. In the 20-min discussions, the perceptions of each profession regarding the education of family medicine trainees, their difficulties, and suggestions were addressed. The contents were recorded in field notes. One-on-one interviews were conducted with the participants every four months to obtain an in-depth perception from each participant. They were interviewed regarding their perceptions of family medicine trainees and the observed changes in their work resulting from the program. Furthermore, the revised points of the educational curriculum were discussed. All interviews were recorded and transcribed verbatim.

### 2.5. Analysis

The quantitative data were analyzed by the student’s t-test, and nonparametric data were analyzed using the Wilcoxon signed-rank test. Categorical data were compared using the chi-square test. The diagnoses of the patients were categorized based on the 10th revision of the International Statistical Classification of Disease and Related Health Problems (ICD-10). The average frequency of each code was defined as the score of the total code numbers divided by the total number of types of codes in each month. The average frequency of each code was compared between April 2020 and March 2021. The statistical significance was set at α = 0.05. The qualitative data were analyzed, including the contents of field notes, one-on-one interviews, and focus groups, based on the grounded theory approach [26]. In the open coding, the analysis of the contents of the interviews and field notes included process and concept coding. The first author read and coded the contents and made the code book for further analysis. The second author also read and coded the contents independently. The first and second authors discussed their coding until a consensus was reached, after which each participant’s interview content was coded. In axial coding, the concepts and themes that emerged in the open coding cycle were refined by revising interview contents, codes, concepts, and themes to clarify the changing processes. This process was performed iteratively until theoretical saturation occurred. Finally, through a discussion among the researchers, a theoretical framework was established.

### 2.6. Ethical Consideration

The participants were informed prior to involvement in the research, and before the start of the interviews, that the data collected would only be used for research purposes. The participants were also informed of the aims of the research, how the data would be disclosed, how their personal information would remain confidential, and that they could withdraw should they choose to do so. The hospital was assured of confidentiality regarding the patients′ information. The information related to this study was posted on the hospital website without disclosing any details concerning the patients. To address any questions regarding this study, the contact information of the hospital representative was also listed on the website. This study was approved by the Unnan City Hospital Clinical Ethics Committee (approval number: 20200001).

## 3. Results

### 3.1. Characteristics of Patients Who Were Cared for by the Medical Trainees and the Participants in the Interviews

The total number of inpatients and outpatients in the care of family medicine residents in April 2020 was 39 and 265, respectively, and in March 2021, 67 and 532, respectively. The average age of admitted patients and outpatient departures was 80; 2-years-old (standard deviation = 13.9) and 63.8-years-old (standard deviation = 20.6) in April 2020, and 79.3-years-old (standard deviation = 17.6) and 65.0-years-old (standard deviation = 22.3) in March 2021 (Table 1). The interviews involved 5 medical educators, 3 family medicine trainees, 13 medical students/residents, 17 nurses, 7 pharmacists, 3 therapists, 3 social workers, 2 clinical technicians, 1 nutritionist, and 3 clerks.

### 3.2. Change in the Range of Diseases

In outpatient departures, the number of ICD codes compared to the total number of codes was 150:347 in April 2020, and 295:1095 in March 2021. In admitted patients, the number of ICD codes compared to the total number of codes was 234:512 in April 2020, and 304:1104 in March 2020. Regarding experiences per code, the timing in March 2021 was statistically more prominent than in April 2020 in both outpatient and admission categories (Table 1).

### 3.3. Score of the Quality of Interprofessional Collaboration from Nurses

The questionnaires regarding interprofessional collaboration were collected from deputy directors of the nursing departments (12/12, 100% collection rate). The score improved significantly in the categories of introducing oneself, politeness to nurses, respect for nurses as members of the medical team, and respecting nurses’ ideas concerning care management (Table 2).

### 3.4. Qualitative Analysis of the Change in Stakeholders

Three themes and nine concepts were extracted using the grounded theory approach. The themes were conflicts with the past, driving unlearning, and organizational change (Table 3). Each theme is explained, based on the relevant concepts, and related quotations.

#### 3.4.1. Conflict with the Past

Each stakeholder had an understanding of their own work and that of other professionals, which impinged on collaboration when introducing family medicine education. The implementation forced them to change their previous routines and professional relationships, which required learning about new aspects of medical education. The stakeholders struggled with the changes, inducing conflicts among them.

##### Acquired Routine

The stakeholders had their own routines for their hospital work. The introduction of family medicine education demanded changes in their workflow. Medical trainees had to learn how to behave as physicians in new inpatient and outpatient departments, acquiring skills such as using electronic medical records and managing patients’ care in collaboration with other professionals.

*I struggled with getting used to this hospital’s system such as medical records and how to prescribe like that. I did not have any opportunity to collaborate with others.*

*(Family medicine trainee 1)*

Nurses had to adjust their work to the new trainees because they came from other institutions with different systems. The nurses had to observe the trainees, which slowed their work and caused them to feel stress.

*Medical education impinged on my work. Medical trainees did not understand the hospital systems at all. So, I had to instruct them to act properly in this hospital. There was help from teaching doctors and medical clerks, but my working speed was slowed down, and our patients might have been inconvenienced.*

*(Nurse 3)*

New applications of family medicine education require support from various departments in hospitals. In the initiation of education, the medical practice was inhibited by the additional work of teaching and instructing trainees. Stakeholders, including trainees, experienced significant stress at the initial stage.

##### Previous Professional Relationships

Each stakeholder had presumptions regarding their relationships with other professionals. Medical trainees retained images of other professionals in hospitals developed through previous hospital experiences, which inhibited collaboration. The trainees felt significant gaps in the relationships among professionals between the present and prior hospitals. One of the family medicine trainees stated:

*In the previous hospital, nurses had more power in decision-making, and I did not strongly insist on my idea. However, here, nurses do not act without decisions. This can be related to rural settings and the education of other professionals in rural hospitals. I was confused with the situation and struggled to discuss with nurses how to care for patients effectively.*

*(Family medicine trainee 3)*

Medical teachers also struggled with the attitudes of the family medicine trainees toward other medical professionals. One of the medical teachers stated:

*The medical trainees are making efforts to take care of patients. However, they tend to wait for nurses’ and other professionals’ comments to improve patients’ care. On the other hand, when changing their decision to respect other professionals’ opinions, they tend to become stubborn. They are struggling to change their attitudes as physicians to fit our setting.*

*(Medical Teacher 1)*

Family medicine trainees struggled with, and were confused about, the differences from previous clinical settings and experienced conflicts regarding how to change their attitudes as physicians.

##### Learning Burden

The stakeholders had to learn family medicine education systems and adjust their attitudes toward family medicine trainees. As there were various limitations regarding what the trainees could do, other professionals had to consider which issues they could consult with them about or discuss with their medical teachers. One participant stated:

*I did not know what the trainees could do in clinical situations and their practical abilities. When I consulted with them, I was told to discuss it with the medical teachers and confused them. For me, medical trainees and medical teachers were both doctors. I did not differentiate the content that could be consulted with medical trainees.*

* (Nurse 8)*

Other professionals confused the trainees’ capacities regarding the ordering of tests. One participant stated:

*The medical trainees sometimes ordered different tests from medical teachers. I did not know the accuracy of their order, so I checked them with their medical teachers every time. Checking added time to my work.*

*(Laboratory technician 2)*

Family medicine trainees also struggled to learn about the culture of the hospital and the broad field of family medicine. One of the trainees stated:

*Family medicine is a broad specialty in medicine. I had to deal with various medical and social problems of patients. It was difficult to learn a lot, including the hospital’s medical conditions and culture.*

#### 3.4.2. Driving Unlearning

The continuous interaction between family medicine trainees and other stakeholders led to mutual understanding, which mitigated the conflicts among them. They were aware of their prior knowledge about interprofessional collaboration, which induced fatigue. Respecting each other’s specializations helped to improve the reconstruction of relationships, driving stakeholders’ motivation to educate family medicine trainees.

##### Notice of Presumption

They noticed their presumptions regarding education and the relationships among professionals in hospitals through various conflicts among stakeholders. By reaching a mutual understanding of one another’s ideas regarding medical care through interprofessional collaboration, they mitigated their stress in family medicine education. One participant stated:

*I had an image of medical education, which should be provided in universities or large hospitals. I considered that education in community hospitals would not be effective and just time-consuming. However, through the interaction with the trainees and their growth, the instruction can improve the hospital’s patient care and activate other medical staff.*

*(Nurse 14)*

With continuous interaction with family medicine trainees, other medical professionals understood various forms of interprofessional collaboration:

*Medical trainees did not have sufficient knowledge and skills to care for patients. They try to learn various things for better care. As a pharmacist, I realized that I could contribute to their progress by teaching medicine to patients. I think that teaching trainees can play a role in interprofessional collaboration.*

*(Pharmacist 2)*

Other medical professionals realized that changing their perceptions regarding medical education and interprofessional collaboration, including education for trainees, could enhance medical care.

##### Functional New Relationship

Establishing a new relationship between the trainees and other professionals made their work more effective and enhanced their interprofessional collaboration, contributing to patient care:

*Now I can have more calls from nurses, pharmacists, and laboratory technicians than before. I could understand that patient conditions change quickly and approach them.*

*(Medical trainee 2)*

Frequent communication among stakeholders improves the detection of changes in patients’ conditions, leading to better patient care. In addition, other professionals realized that patient care was enhanced by the medical trainees. The realization of the effectiveness of family medicine motivated them to collaborate with trainees more. One participant stated:

*The medical trainees learned a lot and can approach patients from various perspectives. This skill is specific and different from that of other professionals. Their profession can be effective in community hospitals, and should be enhanced and used more.*

*(Nurse 7)*

##### Acceptance of Other Cultures

Through experiences of effective collaboration with other professionals, the trainees began to accept feedback from them, especially nurses. The trainees tried to adjust their attitudes regarding interprofessional collaboration and patient care. This is reflected in the following statement:

*The collaboration can drive my performance in patient care. I was persistent in my previous attitudes toward collaboration with nurses. However, I had to understand the culture and environment of medical care in this hospital. By respecting nurses’ suggestions, such as patients’ symptom changes and family perceptions, I can now deal with various patient issues effectively.*

*(Medical trainee 3)*

The nurses noticed the change in the trainees’ attitudes and realized a smooth transition in their collaboration. They also understood that medical trainees had difficulty integrating their previous experiences and altering their activities. A nurse stated:

*The trainees are changing their practices and gradually fitting this hospital’s culture. I understand that they might suffer from changing themselves because they could not help following their previous standards at the beginning of their training. Their practice changes are improving patients’ care. Nurses can change the practices slightly, fitting the trainees’ methods through discussions with them.*

*(Nurse 1)*

The mutual understanding of cultures dependent on clinical experience has established better patient care methods in the community hospital. The process enabled each professional to modify their practices to accommodate others.

#### 3.4.3. Organizational Change

The realization of the effectiveness of family medicine and training improved the quality of interprofessional collaboration and patient care in the community hospital. The change in the hospital’s atmosphere related to family medicine education enabled various professionals to collaborate with the trainees and give them more opportunities to become involved in patients’ medical and social issues, which broadened the trainees’ scope of practice. Furthermore, trainees’ involvement in finding solutions to various issues as well as the support of medical professionals improves their satisfaction and education.

##### Driving Interprofessional Collaboration

Mutual understanding among medical professionals drove their collaboration by respecting each other’s backgrounds. The enhanced collaboration benefits trainees, other professionals, patients, and medical teachers by reducing their stress:

*Initially, there were a lot of accusations from other professionals regarding the trainees’ attitudes and their performances as physicians. I frequently had to deal with the accusations. However, the amounts gradually reduced, and a good reputation appeared. Thanks to them, my stress was reduced, leading to better education and patient care.*

*(Medical Teacher 1)*

In addition to medical care, the medical clerks recognized a change in the trainees’ attitudes toward administrators, which drove interprofessional collaboration. The Medical trainees began to seek advice from medical clerks regarding their non-technical skills. One participant stated:

*The medical trainees were not used to the situation where they got many questions regarding their medical performance related to the insurance systems, which might irritate them. They are getting used to the system and trying to get advice from us. Although they might have pride as physicians, they could change their attitudes to the medical clerks.*

*(Medical clerk 3)*

The trainees’ adjustment in the clinical setting and improved acceptance of other professionals through continual interprofessional collaboration mitigated their challenges.

##### Broadening the Scope of Practice

Through collaboration with various professionals, family medicine trainees exercised skills across the borders of organ-specialist areas, such as approaching patients with multimorbidity. Their approaches to patients motivated other professionals to offer them various challenges to patients’ problems, which has increased trainees’ opportunities to approach more medical issues of patients without consulting other specialists:

*I thought that physicians have only one specialty and approached the diseases of their specialties. I had never offered any approaches to physicians, except for the issues of their specialties. They also consulted about their patients’ problems with other specialists. However, the trainees and family medicine physicians do not. They can approach various patient issues. Although their approach was a bit strange for me at first, their practice is now useful to treat older patients with various medical problems and frailty.*

*(Nurses 3)*

The trainees also recognized the broadening of their scope of practice and felt satisfaction in developing their clinical skills and patient care. One participant stated:

*Now, I can do various things with patients, as I have to learn every day. Nurses and other professionals have begun to ask for more help with patients’ problems. I feel that I may become reliable for other professionals and satisfy the present performance. Now, I am motivated to approach an increasing number of my patients’ problems through learning.*

*(Medical trainees 1)*

##### Educational Culture

Realizing the effectiveness of family medicine training motivated other professionals to educate medical trainees in the community hospital. The initial phase of the application of family medicine education put a burden on various medical professionals, but they realized that through continual education of the trainees, their growth could mitigate their difficulties in clinical situations and provide better care to patients. This is reflected in the following participant statement:

*Endurance is essential for education. I know, but at first, as I have never experienced a family medicine trainee’s education, I was confused and refused it. However, the trainees improved their skills and attitudes toward both patients and me, a nurse. I am working more effectively than in the past. Now, I understand that the trainees’ education is important in community hospitals.*

*(Nurse 13)*

The involvement of various professionals in the trainees’ education drove the education of medical students and residents in the community hospital. The presence of family medicine residents mitigated the learning difficulties of students and residents. One participant stated:

*The family medicine trainees are vital for my learning. As other students and residents might say, although the presence of sophisticated physicians is essential, their presence enables us to imagine our future vividly. I learned concretely and productively in this hospital. This trend can drive students’ learning, and more students can be motivated to come to this hospital.*

*(Medical student 3)*

Developing and driving family medicine education involving various professionals in a community hospital created an educational culture, leading to better education not only for family medicine trainees but also for medical students and residents.

## 4. Discussion

This study demonstrates that family medicine trainees can improve their scope of practice and the competencies of interprofessional collaborations with nurses through education in rural community hospitals. During the implementation process, the trainees and other professionals can experience conflict in various situations due to previously acquired routines. Through continual collaboration with medical professionals, the trainees can progress in their unlearning and understand sociocultural aspects in rural community hospitals, gradually leading to effective collaboration with them. Although some points still exist for revision, other professionals can accept family medicine trainees and provide more opportunities to the trainees, broaden the trainees’ scope of practice, and the trainees can collaborate with other professionals effectively. Furthermore, the trainees’ efforts can build the educational culture of training in rural community hospitals.

Based on the statistics from ICD-10 coding, it is evident that the scope of practice of family medicine has expanded and become more varied. The analysis of the coding of diseases is supported by the questionnaires [27,28,29]. Previous studies have demonstrated that family medicine requires a wide scope of practice, and the trend is more robust in rural settings, which can motivate medical trainees to become family and general physicians [30,31,32]. The broader scope of practice of family and general physicians can improve medical care comprehensiveness, which can address the difficulties of older people in healthcare [31,33,34] and lead to improved mental health and motivation [32]. This study indicates that training in rural community hospitals can broaden the scope of practice and provide sufficient experience for trainees, facilitated by medical educators. The present family medicine education in Japan lacks educational resources, especially in rural areas [17]. Central and local governments should allocate not only healthcare resources but also educational resources such as medical educators and medical residents who are motivated to become family physicians to rural areas. Further investigation can improve family medicine education in rural settings and provide additional evidence regarding the effects of the implementation of family medicine in rural settings on the health outcomes of its patients.

The application of family medicine education can improve working conditions of rural medical professionals and interprofessional collaboration in community hospitals, leading to better education for family medicine trainees. Rural community hospitals lack medical resources, especially physicians, as medical professionals are aging and retiring [19,35,36]. The family medicine education system can recruit physicians from outside rural areas, which is a potential solution to the lack of medical resources in rural settings [37,38,39]. In addition, educational systems can offer rural area students with special quota in medical schools with the condition that they return to their rural areas to serve as family physicians [40]. Furthermore, in family medicine education, interprofessional collaboration is critical for patient care [41]. This study demonstrates that family medicine training can alter medical trainees’ behaviors, leading to better interprofessional collaboration. This change can be facilitated through mutual education between medical professionals, especially nurses [42]. For improved patient care in all medical institutions, interprofessional collaboration is critical, especially in addressing multimorbidity and older patients [25]. Professional commitment in family medicine education can mitigate difficulties caused by the lack of family medicine educators in clinical settings and their attitudes toward patients and communities [42]. Rural family medicine training can provide a solution not only for the lack of healthcare professionals, but also for improved interprofessional collaboration, which should be encouraged nationally by governments. Future studies could investigate the effects of the education of other professionals on family medicine trainees, driving family medicine education in rural contexts.

This study shows the practical process of implementing family medicine education in rural contexts. The initial phase of the implementation triggered various conflicts with other professionals, which can cause an initial rejection, as organizations tend to have inertia [43]. By continuing the education and adjustment of the attitudes of medical trainees and other stakeholders, each professional’s unlearning might progress [44]. Unlearning is vital for changing both individuals and organizations, driven by mutual understanding and reflection on the identities and values of each [44,45]. In this study, other professionals realized that the education of family medicine trainees could enable them to care for patients effectively by increasing their workforce and improving their clinical skills. Furthermore, improved interprofessional collaboration and the establishment of an educational culture could drive family medicine education in rural contexts [46,47]. Enriching educational environments and the commitment of various professionals, including citizens and patients, are critical for the continuity and improvement of family medicine education [48]. This result represents one of the exemplary processes of the implementation of family medicine education in typical rural community hospitals. Based on this result, other rural community hospitals should begin family medicine education for improved health care. Furthermore, to facilitate improved family medicine education, future studies should inquire into the motivation of other professionals for education and monitor the promoters and impediments of the process and the rate of retainment of the medical trainees in rural settings.

### Limitation

One of the limitations of this study is its transferability. As this study was implemented in a rural community hospital, which may lack responsiveness, we collected qualitative and quantitative data from various perspectives. This study can serve to stimulate further investigation into the implementation of family medicine education and, thus, its implementation in other settings. Another limitation was the duration of follow-up. One year of investigation and observation may not reveal significant changes in the attitudes of the participants. To overcome this challenge, we interviewed various professionals and measured multiple scores that indicated changes in the participants. Future studies could show the effects of family medicine education on community hospitals, patients, and various professionals in the long-term.

## 5. Conclusions

Implementing family medicine education in rural community hospitals can improve the trainees’ experiences as family physicians. During the implementation process, trainees and other medical professionals can experience conflict caused by system and culture change, which can be overcome by unlearning, driven through continuous interaction between the two. For the continuity of family medicine education, methods for moderating conflicts and ensuring effective unlearning—based on the effectiveness of family medicine education—should be promoted in community hospitals.

## Figures and Tables

**Table 1:** Patients’ demographics and the numbers and kinds of ICD-10 codes.

	April 2020	March 2021	*p*-Value
Outpatient departure			
Number of patients	265	532	
age, mean (SD)	63.8 (20.6)	65.0 (22.3)	0.796
sex, man, %	45.5	50	0.632
Total code numbers	347	1095	<0.001
Total kinds of codes	150	295	
Average frequency of each code	2.31	3.71	
Admission			
Number of patients	39	67	
age, mean (SD)	80.2 (13.9)	79.3 (17.6)	0.458
sex, man, %	43.6	38.8	0.646
Total code numbers	512	1104	<0.001
Total kinds of codes	234	304	
Average frequency of each code	2.19	3.63	

**Table 2:** Interprofessional collaboration questionnaire.

	Pre		Post	
Question	Average	SD	Average	SD
Introducing oneself at first	4.69	0.12	4.97	0.06
Respect for nurses’ opinions	4.45	0.21	4.78	0.22
Efficient discussion with nurses	4.88	0.13	5	0
Politeness to nurses	4.45	0.12	4.86	0.05
Respect for nurses as members of medical team	4.29	0.14	4.87	0.23
Respecting nurses’ ideas concerning care management	4.3	0.16	4.62	0.11
Explaining treatment plans to nurses	3.83	0.29	4.07	0.23
Answering nurses’ questions clearly	4.3	0.05	4.44	0.24
Convey treatment plans via oral or electronic medical records to nurses	3.91	0.18	4.01	0.376
Apologizing to nurses when they make mistakes	4.49	0.19	4.55	0.04

**Table 3:** The themes and concepts emerging from the processes of change for the stakeholders.

Theme	Concept
Conflicts with the past	Acquired routine
	Previous professional relationship
	Learning burden
Driving unlearning	Notice of presumption
	Functional new relationship
	Acceptance of other cultures
Organizational change	Driving interprofessional collaboration
	Broadening the scope of practice
	Educational culture

## Data Availability

All data generated or analyzed during this study are included in this published article.
